# Prevalence and Trends of HIV, Syphilis, and HCV in Migrant and Resident Men Who Have Sex with Men in Shandong, China: Results from a Serial Cross-Sectional Study

**DOI:** 10.1371/journal.pone.0170443

**Published:** 2017-01-19

**Authors:** Jun Hu, Xu Gu, Xiaorun Tao, Yaosheng Qian, Giridhara R. Babu, Guoyong Wang, Meizhen Liao, Larry Han, Dianmin Kang, Weiming Tang

**Affiliations:** 1 Institute of HIV/AIDS Prevention and Control, Shandong Provincial Center for Diseases Control and Prevention, Shandong, China; 2 Department of Epidemiology, School of Public Health, Weifang Medical College, Shandong, China; 3 Public Health Foundation of India, IIPH-H, Bangalore, India; 4 Project-China, University of North Carolina, Guangzhou, China; National and Kapodistrian University of Athens, GREECE

## Abstract

**Background:**

Migrant men who have sex with men (MSM) have a higher predisposition for HIV transmission. We aimed to determine and compare the prevalence and trends of HIV, Syphilis, and HCV between migrant and resident MSM in Shandong, China.

**Methods:**

A serial cross-sectional study was conducted in eight cities in Shandong, China from 2010 to 2014. The surveys collected information on demographics, HIV-related knowledge, and HIV-related behaviors including the serologic status of HIV, syphilis, and HCV. Bivariate and multivariable logistic regressions were used to determine differences between migrant and resident MSM.

**Results:**

The overall prevalence of HIV among the 15,705 MSM (14120 were resident, 1580 were migrant and 5 were missing) was 2.6%, with an increase of 1.0% in 2010 to 4.4% in 2014. Prevalence of HIV was higher among migrant MSMs (5.5%) compared to resident MSMs (2.3%). Compared to residents, migrants also had higher prevalence of syphilis (7.5% vs 4.9%) and HCV (1.1% vs 0.6%). We found that there was an increase in the proportion of migrant MSM engaging in anal sex [adjusted OR (AOR) = 1.41 in migrants vs 1.12 in residents], condom use during last anal sex (AOR = 1.14 in residents, *P for trend* = 0.32), consistent condom use (AOR = 1.04 residents, *P for trend* = 0.11) and drug use (AOR = 1.51 in migrants and 1.29 among residents). Except in the year 2011, receiving some health services in last year was significant for people who were HIV-positive compared to negative. (*P for trend* <0.05).

**Conclusions:**

Prevalence of HIV increased in resident as well as migrant MSMs. The migrant MSMs had higher STIs compared to resident MSMs and therefore, should be targeted for effective interventions aimed at reducing their risk behaviors. Deeper understanding of the role of migration in health issues is required for combating the persistently high and gradually increasing HIV burden in MSM in China.

## Introduction

Sexually transmitted infections (STIs) including HIV, syphilis and HCV are major global health problems in men who have sex with men (MSM)[1]. Despite the overall decline in HIV incidence, developed nations such have witnessed a rise in incidence among MSM[[2, 3]. There is also evidence of a sustained epidemic of STI continuing in MSM in several other countries[1]. These contrast sharply with the overall increasing prevalence of HIV in MSM in China [3]. In addition, the reports indicate that MSM engaging in high-risk sexual behaviors has changed over a period of time in China[1]. This is fueled by dramatic demographic shifts seen in China, as most of the men migrate from rural to urban areas in search of employment. As a result, internal migration within China is established as a major factor influencing the distribution of the HIV epidemic in China.

Several studies have documented that migrants have high risky sexual behaviors and STIs than non-migrants [4][5]. Male Migrants seek sexual relationships in the area of the workplace; become part of a newer sexual network, which may in part explain the rise in STI incidence[6]. The higher risk of STIs in migrants may be borne out of ignorance about; safe sex practices, available health services for prevention and therefore, are less likely to utilize the STI prevention services. In addition, migrants might also be infected prior to their migration[7]., The accurate data on the quantum of change in the prevalence of STIs over time and documentation on shifts in sexual behaviors in the migrant and resident MSMs are largely unavailable. By examining the trends in HIV/STIs and sexual behaviors in migrant and resident MSM in Shandong, China; this study aims to fill the knowledge gap and aims to help in understanding the requirement for setting policy priorities.

## Methods

### Design

We conducted a serial cross-sectional study using data collected from sentinel surveillance systems in eight different cities between 2010 and 2014 in Shandong, China. The study protocol was standardized and was uniformly implemented in all the surveillance sites. Staff members were trained in a standardized manner using an approved training module. We followed multistage mixed method sampling; wherein, recruitment was done through snowballing using the venues and internet.

### Participants

The included participants were men aged 18 years or older, have had sex with another man during the past year and provided informed consent for participating in the study. In order to avoid duplicate measurements, the individuals who had taken part in the study in the same year were excluded. The snowball sampling comprised of identifying MSM who were eligible and soliciting information from them to recommend others who might be eligible. We used probability proportional to size for selection sampling based on the available samples in each study city. This was supported by a comprehensive list made available by the Centers to Disease Control (CDC); that contained maps of all potential meeting places for sexual partners of MSMs. We categorized these venues into two groups. The first one (Group A) comprised of pubs, discos, tearooms, clubs, bathhouses, and saunas. The second group (B) included places such as parks, public restrooms, and public lawns. The prerequisite numbers of venues in each selected city were selected from both groups using the method of probability proportional to size. In contrast, we used notifications regarding the study in Internet-based sampling; wherein, invitations for participation were sent to the online audience including posting on local MSM community-based organizations. We sought the help of discussion forums of local gay websites and online chat rooms for greater participation.

### Sample size

Based on recommendations of the WHO [8], we estimated that 250 to 400 persons are required to be recruited in each city. The sample size was calculated by using the formula: $n_{i}=\frac{\left(z_{1-\frac{\alpha }{2}}^{2}p(1-p)\right)}{e^{2}},$ where, $z_{1-\frac{\alpha }{2}}^{2}$ = 1.96 at the 95% confidence level; p is the expected proportion of patients with the outcome (e.g. HIV prevalence in Jinan); *e*^2^ is the expected half-width of the interval[5, 9]. Thereby, we recruited 250 in two cities of Heze and Zibo and 400 in other four cities.

### Behavioral measures

Using a structured and pretested protocol, we collected data about demographics, sexual practices, and correlates of HIV and different STIs. We defined *migrant* MSM as those participants who had residency outside of Shandong Province, while resident MSM had residency within Shandong Province. Sexual practices included having intercourse with both male and female partners, frequency of condom use during sex, condom use during last anal or vaginal sex in the past 6 months. Information on commercial anal intercourse, condom use in the past 6 months, and history of injecting drugs (yes or no) were also collected. HIV-related knowledge was evaluated using eight questions. Answering six or more questions correctly (75% or higher) was defined as having adequate HIV-related knowledge. Data on apportionment of free condom/oils, needle exchange, care programs, guidelines, treatment, coordinating and testing for HIV/STIs was collected. No unique identification information was preserved and all the interviews were anonymous.

### Serologic measures

Subsequent to conducting interviews, five milliliters of venous blood was collected from every member for confirming the status of HIV, HCV, and syphilis. For both HIV and HCV, one test positive for respective Enzyme-Linked Immunosorbent Assay (ELISA) test (ELISA-1) was used for screening purpose; while, diagnostic after positive for (ELISA-2). In the case of Syphilis, antibodies were screened utilizing an ELISA test but a Rapid Plasma Reagin (RPR) was used to confirm ELISA positives. All screening and corroborative tests were carried out in the research facilities at the CDC, Shandong province.

### Data analysis

Trained data entry operators double entered the data using the software EpiData 3.0; with necessary logic checks were performed centrally. We used SAS (SAS Institute. version 9.4.) for all the statistical analysis. Descriptive analyses were conducted to determine the distribution of socio-demographic factors of participants and to calculate the prevalence of HIV, syphilis, and HCV. Cochran-Armitage Trend tests were conducted to examine trends in HIV, syphilis, HCV prevalence, as well as the behaviors of the participants. Bivariate and multivariable logistic regressions (odd ratios (OR) and corresponding 95% confidence intervals (CI) were conducted to test the trends over time. Chi-square tests were used to test differences of factors associated with HIV/STIs between HIV positive and negative tests each year.

### Ethical statement

"Written informed consent was obtained from each participant prior to the interview and blood collection. The study was carried out in accordance with approved guidelines. The study protocol, inform contents and procedures were reviewed and approved by the Institutional Review Board of the Shandong Centers for Disease Control and Prevention (15–1221). The anonymous data (S1 Dataset) and informed consents were kept confidential.

## Results

### Demographic characteristics

Overall, the data comprised of 15,705 participants from 8 study sites between 2010 and 2014. During the study period, each city met the minimum size requirement. For example, among a total of 3060 participants in 2014, 321 were in Heze, 329 in Zibo, equivalent to 400 or more in Binzhou, Yantai, Jinan, Jinin, Liaocheng and Qingdao. Among the participants, 89.9% were residents of Shandong and 10.1% were migrants. From 2010 to 2014, the proportion of migrants decreased from 11.4 to 8.9% (*P*<0.05). The study population comprised of predominately younger people (62% of residents and 64% of migrants were aged between 20 and 29). A majority of the residents 9419 (66.7%), as well as migrants 1147 (72.7%), were never married. More than a third had a college education or higher (6174, 39.3%). Compared to migrants, resident MSM was more likely to find partners through a park or public restrooms (27.6% for residents and 17.4% for migrants) (Table 1).

**Table 1:** Demographic characteristics of MSM recruited between 2010and 2014 in Shandong (N = 15705).

Characteristics	2010		2011		2012		2013		2014		Total	
	(N = 3087)		(N = 3192)		(N = 3202)		(N = 3164)		(N = 3060)		(N = 15705)	
	Resident(%)	Migrant(%)	Resident (%)	Migrant (%)	Resident (%)	Migrant (%)	Resident (%)	Migrant (%)	Resident (%)	Migrant (%)	Resident (%)	Migrant (%)
**Percent of the class**	88.6	11.4	89.4	10.6	89.9	10.1	90.8	9.2	91.1	8.9	89.9	10.1
**Age(years)**												
<20	4.4	6.3	6.0	8.3	5.0	5.0	3.6	3.4	6.5	4.8	5.1	5.7
20–29	74.2	71.6	61.1	62.8	55.7	56.3	59.8	61.7	58.5	65.9	61.8	63.8
30–39	16.1	17.3	23.7	20.6	29.8	30.7	25.9	23.3	26.3	22.3	24.4	22.7
40–49	4.6	3.7	7.8	7.1	7.7	6.5	8.6	8.9	6.4	7.0	7.0	6.5
≥50	0.7	1.1	1.4	1.2	1.8	1.5	2.1	2.7	2.3	0.0	1.7	1.3
**Marital status**												
Single	75.8	82.1	64.4	73.4	58.1	61.3	67.0	68.8	68.7	77.3	66.7	72.7
Married	20.6	16.2	31.8	22.2	38.6	32.5	27.8	26.4	26.4	16.5	29.2	22.7
Widowed	3. 6	1.7	3.8	4.4	3.3	6.2	5.2	4.8	4.9	6.2	4.1	4.6
**Education**												
Senior High School or lower	59.6	66.8	60.6	67.0	61.2	64.4	62.8	56.8	58.3	44.3	60.7	60.6
College or Above	40.4	33.2	39.4	33.0	37.8	35.6	37.2	43.2	41.7	55.7	39.3	39.4
**Venues**												
Pub, Disco, Tearoom, or Club	26.3	29.6	23.6	21.5	18.7	21.3	26.8	24.5	28.5	16.5	24.7	22.9
Bathhouse	8.9	13.6	15.5	21.2	22.6	22.9	13.0	12.1	7.0	7.7	13.5	15.9
Park/Public Restroom	24.8	14.5	26.3	14.8	32.0	21.8	25.0	16.6	29.6	20.1	27.6	17.4
Internet	13.8	7.9	9.8	4.7	6.1	4.9	8.8	3.4	15.3	22.7	10.7	8.4
Others	26.2	34.4	24.8	37.8	20.6	29.1	26.4	43.4	19.6	33.0	23.5	35.4

### Prevalence trends of HIV, syphilis, and HCV

The overall prevalence of HIV increased from 1% to 4.4% during 2010–14, (*P*<0.01); it rose from 0.9 to 4.0% (*P*<0.01) in residents and 2.3 to 8.9% in migrants (*P*<0.01). The average prevalence of HIV was 2.6% (414). Both bivariate and multivariable models indicated increasing trend of HIV prevalence over years (unadjusted Odds Ratio (uOR) = 1.42, 95% CI: 1.31–1.55, Adjusted Odds Ratio (AOR) = 1.40, 95% CI: 1.28–1.52 in residents, while uOR = 1.43, 95%CI: 1.22–1.69, AOR = 1.44, 95%CI: 1.22–1.70 in migrants). In addition, the infection rate of HIV in migrants was much higher than in residents (Table 2).

**Table 2:** HIV, syphilis, and HCV prevalence from 2010 to 2014 in Shandong, 2010–2014 (N = 15705).

Variables	2010 (%)	2011 (%)	2012 (%)	2013 (%)	2014 (%)	*P* for Trend	Crude OR (95%CI)		Adjusted OR* (95%CI)		*P* for adjusted model
**HIV**											
Resident	0.9	1.8	1.8	3.1	4.0	<0.01	1.42	(1.31–1.55)	1.40	(1.28–1.52)	<0.01
Migrant	2.3	3.3	5.6	8.9	8.4	<0.01	1.43	(1.22–1.69)	1.44	(1.22–1.70)	<0.01
All	1.0	1.9	2.2	3.6	4.4	<0.01	1.41	(1.31–1.52)	1.39	(1.29–1.49)	<0.01
**Syphilis**											
Resident	4.4	4.5	5.1	5.9	4.4	0.28	1.03	(0.98–1.09)	1.01	(0.95–1.07)	0.77
Migrant	6.5	8.3	7.4	8.2	7.3	0.71	1.02	(0.90–1.17)	1.04	(0.90–1.19)	0.62
All	4.7	4.9	5.3	6.1	4.5	0.33	1.03	(0.98–1.08)	1.01	(0.96–1.06)	0.83
**HCV**											
Resident	0.4	0.7	0.8	0.7	0.6	0.44	1.06	(0.91–1.23)	1.04	(0.90–1.21)	0.58
Migrant	2.0	0.9	1.6	0.0	1.1	0.14	0.77	(0.54–1.10)	0.73	(0.50–1.06)	0.10
All	0.6	0.7	0.9	0.6	0.7	0.97	1.00	(0.88–1.15)	0.99	(0.86–1.13)	0.84

Note:

* Multivariate models were adjusted for the following variables: age, marital status (ref = married) and education.

Prevalence of syphilis and HCV did not change much. Despite no change in the overall prevalence of syphilis (5.1%); the prevalence was consistently higher in migrants compared to residents (6.5% to 8.3% in migrants, *P* = 0.71 vs. 4.4% to5.9% in residents, *P* = 0.28). Less than 1% of participants were HCV positive, with higher rates in migrants (0% to 2%, *P* = 0.14) compared to residents (0.4% to 0.8%, *P* = 0.44). Bivariate and multivariable analyses indicated that there was no significant change in syphilis and HCV prevalence.

### Behaviors for MSM

Overall, 77.6% of residents and 88.7% of migrants admitted that they had engaged in anal sex in the past 6 months. The increase was significant in residents (72.4% in 2010 to 82.1% in 2012, *P*<0.01) as well in migrants (80.5% in 2010 to 94.9% in 2014, *P*<0.01). (AOR = 1.12, 95%CI: 1.01–1.15 in residents, while AOR = 1.41, 95%CI: 1.25–1.60 in migrants). The use of condoms during last anal intercourse with other men increased from 76.8% in 2010 to 85.3% in 2013 for residents (AOR = 1.14, 95%CI: 1.10–1.17). About 51.6% of residents and 44.1% of migrants consistently used condoms during anal sex in the last 6 months. This proportion increased from 49.3% in 2011 to 53.9% in 2014 (AOR = 1.04, 95%CI: 1.02–1.07) in residents and no significant increase was seen in migrants. The proportion of consistent condom use was lower in migrants than in residents.

About one quarter of participants admitted engaging in vaginal intercourse with female, ranged between 22.8% (2010) to 32.7% (in 2012) and 22.9% (in 2014). This proportion changed little and significantly decreased only in the resident (AOR = 0.93, 95%CI: 0.90–0.99). Few (1.5% of residents and 3.6% of migrants) participants used drugs; the proportion increased (0.6% in 2010 to 2.4% in 2012, AOR = 1.29, 95%CI: 1.17–1.43 in both the residents and migrants (0.3% in 2010 to 5.6% in 2012, AOR = 1.51, 95%CI: 1.23–1.86) (Table 3).

**Table 3:** Behaviors of MSM in Shandong, China, 2010–2014 (N = 15705).

Variables	2010 (%)	2011 (%)	2012 (%)	2013 (%)	2014 (%)	*P* for Trend	Crude OR (95%CI)		Adjusted OR* (95%CI		*P* for Adjusted Model
**Engaged in anal sex in the last 6 months**											
Resident	72.4	74.9	82.1	77.4	80.9	<0.01	1.12	(1.01–1.15)	1.12	(1.09–1.15)	<0.01
Migrant	80.5	88.7	90.5	91.1	94.9	<0.01	1.40	(1.24–1.58)	1.41	(1.25–1.60)	<0.01
All	73.3	76.3	83.0	78.6	82.2						
**Used condom during last anal intercourse with male**											
Resident	76.8	82.4	83.6	85.3	84.1	<0.01	1.13	(1.09–1.16)	1.14	(1.10–1.17)	<0.01
Migrant	75.9	70.4	81.7	76.6	76.5	0.32	1.04	(0.96–1.13)	1.06	(0.97–1.15)	0.20
All	76.7	81.1	83.4	84.5	83.4						
**Consistent used condom during anal sex in the last 6 months**											
Resident	51.3	49.3	50.0	53.6	53.9	<0.01	1.04	(1.02–1.06)	1.04	(1.02–1.07)	<0.01
Migrant	44.5	38.6	43.0	48.3	47.1	0.11	1.06	(0.99–1.14)	1.06	(0.98–1.34)	0.16
All	50.6	48.2	49.3	53.1	53.3						
**Engaged in commercial anal sex with male in the last 6 months**											
Resident	9.8	11.8	20.2	11.2	9.3	0.39	0.98	(0.95–1.02)	0.98	(0.95–1.02)	0.25
Migrant	28.0	27.1	24.6	22.4	17.7	<0.01	0.87	(0.80–0.95)	0.94	(0.86–1.02)	0.15
All	11.9	13.4	20.6	12.2	10.1						
**Used condom at last commercial anal intercourse**											
Resident	71.5	73.9	87.0	90.2	81.2	<0.01	1.30	(1.17–1.43)	1.31	(1.18–1.45)	<0.01
Migrant	74.0	59.3	86.8	87.7	70.8	0.11	1.18	(0.99–1.40)	1.15	(0.96–1.39)	0.14
All	72.1	70.8	87.0	89.8	79.6						
**Consistent used condom during commercial anal sex in the last 6 months**											
Resident	32.1	27.2	31.8	43.0	30.8	0.07	1.08	(0.99–1.16)	1.07	(0.99–1.16)	0.11
Migrant	29.5	28.6	39.5	58.5	25	0.07	1,16	(0.99–1.35)	1.18	(0.99–1.41)	0.06
All	31.4	27.5	32.7	45.7	29.9						
**Engaged in vaginal sex in the last 6 months**											
Resident	22.9	30.1	33.1	23.1	23.0	<0.01	0.96	(0.94–0.99)	0.93	(0.90–0.96)	<0.01
Migrant	22.2	20.9	29.3	24.0	22.0	0.64	1.02	(0.94–1.11)	1.00	(0.90–1.10)	0.94
All	22.8	29.2	32.7	23.2	22.9						
**Used condom during last vaginal intercourse**											
Resident	53.5	56.8	54.8	59.0	61.7	<0.01	1.08	(1.03–1.32)	1.10	(1.04–1.16)	<0.01
Migrant	61.0	60.6	44.9	53.6	65.0	0.89	0.99	(0.85–1.15)	1.05	(0.88–1.25)	0.59
All	54.3	57.2	53.9	58.4	62.1						
**Consistent used condom during vaginal sex in the last 6 months**											
Resident	20.7	38.0	23.3	22.1	34.5	0.17	1.04	(0.98–1.10)	1.05	(0.99–1.11)	0.09
Migrant	41.6	30.0	18.7	21.4	46.7	0.77	0.98	(0.83–1.15)	1.00	(0.84–1.19)	0.99
All	23.0	37.5	22.9	22.1	35.5						
**Used drug in lifetime**											
Resident	0.6	0.6	2.4	2.0	1.8	<0.01	1.23	(1.17–1.44)	1.29	(1.17–1.43)	<0.01
Migrant	0.3	2.9	5.6	4.1	5.5	<0.01	1.43	(1.17–1.75)	1.51	(1.23–1.86)	<0.01
All	0.6	0.9	2.7	2.2	2.1						

Note:

* Multivariate models were adjusted for the following variables: age, marital status (ref = married), education.

### Related factors of HIV infection

Anal sex in the past 6 months was associated with HIV infection in three years (2011,2013,2014). Use of condoms during last anal sex and consistent condom use in the last six months were negatively related to HIV infection in 2010 and 2012. Consistent condom use during commercial anal sex was also negatively associated with HIV infection in 2010, 2013, and 2014. Receiving any kind of HIV-related services in the past year was negatively associated HIV infection in 2011 (Table 4).

**Table 4:** HIV-related knowledge, services, and behaviors for HIV negative and positive MSM in Shandong, 2010–2014 (N = 15705).

Variables	2010		2011		2012		2013		2014		
**Engaged in anal sex in the last 6 months**											
HIV-	73.2	(2213/3019)	76.1	(2367/3111) ^a^	82.8	(2569/3104)	78.0	(2376/3046) ^a^	81.6	(2388/2925) ^a^	<0.01
HIV+	86.2	(25/29)	88.3	(53/60)	91.6	(65/71)	95.6	(109/114)	94.1	(127/135)	0.04
**Used condom during last anal intercourse with male**											
HIV-	76.8	(2314/3012)	81.4	(2522/3100)^a^	83.4	(2589/3106)	84.9	(2574/3033)^a^	84.1	(2450/2915)^a^	<0.01
HIV+	63.3	(19/30)	67.0	(40/58)	85.9	(61/71)	74.3	(84/113)	68.7	(92/134)	0.88
**Consistent used condom during anal sex in the last 6 months**											
HIV-	56.6	(1527/3020)	48.6	(1509/3108)^a^	49.2	(1529/3105)	53.46	(1629/3047)^a^	54.2	(1582/2919)^a^	<0.01
HIV+	44.8	(13/29)	31.0	(18/58)	50.7	(36/71)	43.0	(49/114)	33.6	(45/134)	0.40
**Engaged in commercial anal sex with male in the last 6 months**											
HIV-	11.8	(355/3002)	13.3	(413/3102)	20.6	(635/3083)	12.1	(368/3039)	9.9	(289/2924)	0.01
HIV+	19.4	(6/29)	18.3	(11/60)	22.5	(16/71)	15.0	(17/113)	14.2	(19/134)	0.19
**Used condom during last commercial anal intercourse**											
HIV-	72.2	(255/353)	70.0	(289/413)^a^	86.8	(546/629)	90.4	(329/364)	80.3	(232/289)	<0.01
HIV+	166.7	(4/6)	100	(12/12)	93.8	(15/16)	76.5	(13/17)	68.4	(13/19)	0.15
**Consistent used condom during commercial anal sex in the last 6 months**											
HIV-	30.9	(109/353)	26.6	(110/413) ^a^	31.8	(202/630) ^a^	44.75	(162/362)	30.1	(87/289)	0.01
HIV+	50.0	(3/6)	63.6	(7/11)	68.8	(11/16)	64.7	(11/17)	26.3	(5/19)	0.08
**Engaged in vaginal sex in the last 6 months**											
HIV-	22.8	(690/3027)	29.3	(914/3120)	32.8	(1019/3107)	23.3	(709/3046)	22.9	(669/2923)	0.02
HIV+	22.6	(7/31)	24.2	(15/62)	26.8	(19/71)	20.2	(23/114)	23.0	(31/135)	0.73
**Used condom during last vaginal intercourse**											
HIV-	54.9	(374/681)	57.6	(518/900)	54.1	(539/997)	58.5	(413/706)	62.4	(415/665)	0.01
HIV+	14.3	(1/7)	33.3	(5/15)	47.4	(9/19)	56.5	(1323)	54.8	(17/31)	0.03
**Consistent used condom during vaginal sex in the last 6 months**											
HIV-	23.2	(159/685)	37.9	(343/905)	23.1	(233/1010)	21.9	(155/707)	35.1	(233/664)	0.46
HIV+	14.3	(1/7)	13.3	(2/15)	11.1	(2/18)	26.1	(6/23)	45.2	(14/31)	<0.01
**Used drug in lifetime**											
HIV-	0.6	(17/3042)	0.9	(27/3128)	2.6	(83/3131)	2.2	(68/3050)	2.2	(63/2925)	<0.01
HIV+	0.0	(0/32)	1.6	(1/62)	7.0	(5/71)	1.8	(2/114)	0.7	(1/135)	0.49
**Correct HIV-related knowledge**											
HIV-	83.3	(2533/3042)	87.7	(2744/3128)	86.5	(2707/3131)	90.6	(2763/3050)	88.6	(2592/2925)	<0.01
HIV+	84.4	(27/32)	85.5	(53/62)	87.3	(62/71)	95.6	(109/114)	88.9	(120/135)	0.18
**Being received any kind of HIV-related services in last year**											
HIV-	75.3	(2290/3042) ^a^	70.0	(2188/3128)	78.1	(2446/3131) ^a^	77.9	(2375/3050) ^a^	80.9	(2367/2925) ^a^	<0.01
HIV+	56.3	(18/32)	62.9	(39/62)	66.2	(47/71)	67.5	(77/114)	60.7	(82/135)	0.87

Note:

^a^ represents statistical significance (*P*<0.05).

## Discussion

Our study assessed the predominance, and patterns in HIV and different STIs in the migrant and resident MSMs of Shandong, China. The results extend the available evidence by using a serial cross-sectional design and evaluating MSM across multi-center study design. We report that STIs are highly prevalent in migrant MSM populations compared to resident MSM, with an increasing trend of HIV prevalence in both the migrants and residents. In addition, the prevalence of high-risk sexual behaviors, namely engaging in anal sex and consistent condom use (excluding migrants), increased in both subgroups.

The overall prevalence of HIV in Shandong MSM was 2.6%, an increase from figures reported in two studies conducted in 2008 and 2011[9, 10]. However, the prevalence of HIV in Shandong MSM is considerably lower than in many other provinces or cities in China. For example, the prevalence of HIV was 7.7% among Beijing MSM in 2014, which is consistent with results from a meta-analysis in 2009[10]. Internationally, the pooled HIV prevalence in MSM ranges from 3% (95% CI 2.4%-3.6%) in the Middle East and North Africa region to 25.4%(21.4%-29.5%) in the Caribbean[1]. Although the overall prevalence of HIV was low in Shandong, there has been a substantial increase in HIV, with a doubling of the prevalence among MSM since 2010. To reverse this trend, comprehensive strategies are targeted at increasing HIV testing, linkage to care, and retention in care are needed.

Our study indicated that the prevalence of HIV/STIs was higher in migrant MSM than in resident MSM. Similar evidence is available from other studies suggesting higher odds of acquiring or transmitting HIV/ STIs in migrants compared to general population.[11, 12] This finding may be due to several reasons. First, migrant MSM is less likely to have a stable sexual partner and may have multiple partners, increasing the risk for HIV/STIs[1]. Infected migrants may also serve as a bridge population for HIV/STIs transmission when they return to their area of residence[13]. Second, migrants in China are mainly from rural or poor towns[14], often poorly educated and working in low-paid jobs[15]. Lower levels of education attainment may make them less aware of HIV risk and safe sex practices[16]. Third, social isolation can increase the migrant’s the risk of acquiring HIV/STIs[17]. HIV diagnosis through targeted HIV testing campaigns can help reduce the risk of onward sexual transmission[16, 18].

In contrast with available evidence in China, we found comparatively lower proportion (about 49.1% overall) anal intercourse without using condoms[18, 19]. Not using the condom is a definitive risk factor for HIV; and hence partly explains the lower prevalence of HIV in Shandong. We also observed an increasing trend of drug use during the study period; with migrants affected nearly twice as residents. Since Drug use predisposes MSM for higher risk for HIV/STIs[20], efforts should be undertaken to address the problem.

Despite the strength of being a large study with serial cross-sectional design, there were also some limitations. Firstly, data collection was relayed on retrospective self-reporting and this may lead to recall bias. Second, the sensitivity of sexually related questions could lead to reporting bias. Third, we were limited by the unavailability of response rates in our study; and, the effect of mobility of population between cities within Shandong was not available. Fourth, we did not evaluate the proportion of the participants who were selected in multiple years, and if HIV positive persons are sampled in multiple years; this might explain the increase in prevalence. However, one important purpose of Chinese surveillance system is to identify new HIV cases and to report identified cases for further action. Since the proportion of HIV-positive persons sampled in multiple years was consistently low, its influence on the overall trend is minimal.

## Conclusion

In conclusion, STIs are highly prevalent among migrant MSM compared to residents. Engaging in unprotected anal sex in migrants might be the driver of the high STIs in Shandong. The migrant MSM should be targeted for effective interventions aimed at reducing their risk behaviors. Deeper understanding of the role of migration in health issues is required for combating the persistently high and gradually increasing HIV burden in Men who have sex with Men in China.

## Supporting Information

S1 Dataset (XLSX)
